# Association between metabolic syndrome and multiple lesions of intracranial atherothrombotic stroke: a hospital-based study

**DOI:** 10.1186/s12933-015-0272-6

**Published:** 2015-08-14

**Authors:** Kazuhiko Kotani, Noriko Satoh-Asahara, Takuya Nakakuki, Hajime Yamakage, Akira Shimatsu, Tetsuya Tsukahara

**Affiliations:** Division of Preventive Medicine, Clinical Research Institute, National Hospital Organization, Kyoto Medical Center, Kyoto, Japan; Division of Community and Family Medicine, Jichi Medical University, Shimotsuke, Japan; Department of Clinical Laboratory Medicine, Jichi Medical University, Shimotsuke, Japan; Division of Diabetic Research, Clinical Research Institute, National Hospital Organization, Kyoto Medical Center, Kyoto, Japan; Department of Neurosurgery, Hikone Municipal Hospital, Hikone, Japan; Department of Neurosurgery, National Hospital Organization, Kyoto Medical Center, Kyoto, Japan

**Keywords:** Insulin resistance, Obesity, Triglyceride, HDL-cholesterol, Dyslipidemia, Ischemic stroke

## Abstract

**Background:**

With the increasing trend of metabolic syndrome (MetS) and atherothrombotic stroke (which can manifest as stroke lesion multiplicity), studies on the association between MetS and the clinical aspects of atherothrombotic stroke are of great interest. The present study aimed to investigate the association between MetS and multiple atherothrombotic strokes in patients with intracranial atherothrombotic stroke.

**Methods:**

A retrospective study based on medical charts was conducted among patients (n = 202: 137 men/65 women) who were symptomatically admitted to the hospital with the first-ever atherothrombotic stroke. For the occurrence of multiple lesions of stroke, odds ratio [OR: 95 % confidence interval (CI)] of MetS or its respective components was calculated using logistic regression models.

**Results:**

Fifty-one percent of the men and 38 % of women with stroke presented multiple regions. MetS was a significant factor that was associated with an increased risk of multiple regions in women [OR 4.3 (95 % CI 1.4–13.5)], but not in men. According to the components of MetS, dyslipidemia was a significant factor that was positively associated with multiple regions in both men [OR 2.0 (95 % CI 1.1–3.7)] and women [OR 3.2 (95 % CI 1.1–9.1)].

**Conclusion:**

MetS may be pathophysiologically associated with intracranial atherothrombotic stroke multiplicity in women in particular. Future studies are warranted to confirm the findings.

## Background

Metabolic syndrome (MetS) consists of metabolic abnormalities, such as obesity, high blood pressure (BP), hyperglycemia and dyslipidemia (high triglyceride (TG)/high-density lipoprotein cholesterol (HDL-C)) [1, 2]. MetS is considered to be a risk factor of vascular diseases [1, 2]. Therefore, the elucidation of the clinical relevance of MetS in vascular medicine is of importance.

Stroke is one of the vascular diseases, and a better understanding of the pathophysiology of stroke is crucial given its frequency and global socio-medical burden [3, 4]. There has been evidence of a positive association between MetS and the prevalence/morbidity of stroke [5–10]. Of note, intracranial atherothrombotic stroke has gradually increased among ischemic stroke patients with the epidemic of MetS in Japan [11]. In fact, a few studies have reported a positive association between MetS and atherothrombotic stroke [7, 12–14]. With the growing concern of studies for atherothrombotic stroke [11], the association between MetS and the clinical aspects of atherothrombotic stroke should be further explored.

Atherothrombosis has a biological characteristic to lead to multi-bed vascular disorders [15]. Carotid atherosclerosis, reflective of generalized atherosclerotic manifestations, is reported to increase the risk of vascular events in relation to MetS [16]. It is of interest to study the association between MetS and atherothrombotic stroke legion multiplicity; however, there is a paucity of such data. Therefore, the present study aimed to investigate the association between MetS and multiple atherothrombotic strokes in in-patients who were admitted to the hospital with the first-ever stroke.

## Methods

We conducted a retrospective study through an analysis of medical charts (from 2003 to 2006) of patients who were admitted to our hospital with defined intracranial atherothrombotic stroke. The included patients were limited to those with a diagnosis of the first-ever symptomatic stroke. The diagnosis of atherothrombotic stroke was made according to the diagnostic criteria of the National Institute of Neurological Disorders and Stroke by neurological specialists [17]. That is, it was diagnosed in cases with infarcts greater than 1.5 cm in diameter of a major brain and/or branch cortical artery on imaging (a computed tomography and/or magnetic resonance imaging) as possible origins under clinical symptoms of neurological deficits [17]. Strokes in patients with a source of embolus, such as atrial fibrillation, moderate-to-severe valvular heart disease or intra-carotid/cardiac thrombus, were excluded as having a definite or possible cardioembolic stroke [17]. Strokes in patients with an undetermined etiology despite an extensive evaluation were also excluded [17]. The study was approved by the Ethics Committee of Kyoto Medical Center.

A total of 202 patients (137 men and 65 women) were enrolled in the study. The clinical data on the components of MetS and stroke multiplicity in the patients was collected based on the description of the medical records. Like the National Cholesterol Education Program Adult Treatment Panel III [1] (the criterion level for obesity and low HDL-C was modified for Japanese people [2, 18]), the patients were diagnosed as having MetS when the patients had at least three components of MetS. The components were obesity (a body mass index ≥25 kg/m^2^ [2] ), high BP (systolic BP ≥130 mmHg, diastolic BP ≥85 mmHg and/or the use of antihypertensive drugs), hyperglycemia (fasting plasma glucose ≥110 mg/dL and/or the use of glucose-lowering drugs), high TG (fasting serum triglyceride ≥150 mg/dL and/or the use of triglyceride-lowering drugs) and low HDL-C (fasting serum HDL-C <40 mg/dL [18] ). The components of MetS were according to the laboratory data and/or the description in medical records (e.g., if glucose intolerance was clearly described in the charts, it was treated as being within the hyperglycemic criteria).

The differences between the groups were examined by an independent *t* test or the Chi square test. For the outcome variable (having multiple lesions of stroke), odds ratio [OR: 95 % confidence interval (CI)] of the explanatory variables (MetS and its components) was calculated using crude and adjusted logistic regression model analyses. In the logistic regression models, high TG and low HDL-C were treated as a dyslipidemic component because few patients had low HDL-C (this is often seen in Japanese people) and the potential collinearity of low HDL-C with high TG (which mirrors the biological nature). The statistical analyses were also performed according to gender. A P value < 0.05 was considered to be statistically significant.

## Results

Table 1 shows the clinical characteristics of study patients. In men, 51 % of the stroke cases presented multiple regions, while 38 % of women presented multiple regions. There was a significantly higher prevalence of obesity and hyperglycemia in women than in men. In men, the group with multiple legions had a significantly higher prevalence of dyslipidemia (high TG in particular) than the group with a single region. In women, the group with multiple legions had a significantly higher prevalence of dyslipidemia (high TG in particular) and MetS than the group with a single region.

**Table 1:** Clinical characteristics of the patients

	Men			P1	Women			P2	P3
	All	Single	Multiple		All	Single	Multiple		
Number, n	137 (100 %)	67 (49 %)	70 (51 %)	–	65 (100 %)	40 (62 %)	25 (38 %)	–	0.09
Age (years)	68 ± 9	67 ± 11	69 ± 7	0.17	70 ± 10	70 ± 12	69 ± 7	0.62	0.20
Obesity	28 (20 %)	14 (21 %)	14 (20 %)	0.90	24 (37 %)	12 (30 %)	12 (48 %)	0.14	0.01*
High BP	101 (74 %)	49 (73 %)	52 (74 %)	0.88	41 (63 %)	24 (60 %)	17 (68 %)	0.52	0.12
Hyperglycemia	64 (47 %)	26 (39 %)	38 (54 %)	0.07	42 (65 %)	23 (58 %)	19 (76 %)	0.13	0.02*
High TG/low HDL-C	70 (51 %)	28 (42 %)	42 (60 %)	0.03*	33 (51 %)	16 (40 %)	17 (68 %)	0.03*	0.97
High TG	66 (48 %)	26 (39 %)	40 (57 %)	0.03*	31 (48 %)	14 (35 %)	17 (68 %)	0.01*	0.94
Low HDL-C	7 (5 %)	3 (4 %)	4 (6 %)	0.74	7 (8 %)	3 (8 %)	4 (16 %)	0.28	0.14
MetS	25 (18 %)	10 (15 %)	15 (21 %)	0.33	19 (29 %)	7 (18 %)	12 (48 %)	0.01*	0.08

Independent *t*-test, P1: single vs. multiple in men, P2: single vs. multiple in women, P3: all men vs. all women.

*BP* blood pressure, *TG* triglyceride, *HDL* high-density lipoprotein, *MetS* metabolic syndrome.

Table 2 shows the OR (95 % CI) of MetS for multiple lesions. Crude and age-adjusted logistic regression model analyses revealed that MetS was a significant factor (approximately 4-fold) that was positively associated with multiple regions in women, but not men.

**Table 2:** Odds ratio (95 % confidence interval) of MetS for multiple lesions

Variable	All	Men	Women
Crude	2.1 (1.1–4.2, p = 0.03*)	1.6 (0.6–3.8, p = 0.32)	4.3 (1.4–13.5, p = 0.01*)
Age-adjusted	2.2 (1.1–4.3, p = 0.03*)	1.7 (0.7–4.2, p = 0.24)	4.3 (1.4–13.4, p = 0.01*)

Table 3 also shows the OR (95 % CI) of each component of MetS for multiple lesions. The crude and multivariate-adjusted logistic regression model analyses revealed that dyslipidemia was a significant factor (approximately 2- to 3-fold) that was positively associated with multiple regions in both men and women.

**Table 3:** Odds ratio (95 % confidence interval) of each component of MetS for multiple lesions

Variable	All	Men	Women
Crude			
Age (years)	1.0 (1.0–1.0, p = 0.52)	1.0 (1.0–1.1, p = 0.14)	1.0 (0.9–1.0, p = 0.62)
Obesity	1.2 (0.6–2.2, p = 0.62)	0.9 (0.4–1.8, p = 0.77)	2.2 (0.8–6.1, p = 0.15)
High blood pressure	1.2 (0.7–2.3, p = 0.49)	1.4 (0.7–2.8, p = 0.31)	1.4 (0.5–4.1, p = 0.52)
Hyperglycemia	1.8 (1.0–3.1, p = 0.04*)	1.5 (0.8–2.8, p = 0.16)	2.3 (0.8–7.1, p = 0.13)
High TG/low HDL-C	2.3 (1.3–4.1, p < 0.01*)	2.0 (1.1–3.7, p = 0.02*)	3.2 (1.1–9.1, p = 0.03*)
Multivariate-adjusted			
Age, years	1.0 (1.0–1.0, p = 0.33)	1.0 (1.0–1.1, p = 0.05)	1.0 (0.9–1.0, p = 0.56)
Obesity	1.1 (0.6–2.2, p = 0.69)	1.0 (0.4–2.3, p = 0.95)	1.9 (0.6–5.8, p = 0.27)
High blood pressure	1.2 (0.6–2.3, p = 0.54)	1.0 (0.4–2.2, p = 0.99)	1.4 (0.5–4.3, p = 0.56)
Hyperglycemia	1.7 (0.9–3.1, p = 0.06)	1.9 (0.9–3.9, p = 0.07)	2.2 (0.7–7.5, p = 0.20)
High TG/low HDL-C	2.5 (1.4–4.4, p < 0.01*)	2.6 (1.2–5.4, p = 0.01*)	3.0 (1.0–8.9, p = 0.046*)

Multivariate-adjusted model: age, obesity, high blood pressure, hyperglycemia and high TG/low HDL-C were entered into the model as variables for multiple lesions.

*MetS* metabolic syndrome, *TG* triglyceride, *HDL* high-density lipoprotein.

## Discussion

In the present study, MetS showed a significant positive association with multiple lesions of intracranial atherothrombotic stroke in women, but not men, who were in-patients admitted to the hospital with the first-ever stroke. The observation that MetS may be an underlying entity for the formation of intracranial stroke multiplicity with a possible gender bias is new information. Atherothrombotic stroke is considered a treatable target [11], while in patients suffering from multiple lesions of stroke, it may lead to multiple neurological and/or cognitive dysfunctions [19, 20]. Thus, the treatment of MetS can be significant for preventing atherothrombotic stroke multiplicity and subsequent dysfunctions in women in particular.

A positive association between MetS and atherothrombotic stroke has been reported [7, 12–14], and the etiopathological mechanisms of atherothrombosis (e.g., via vasomotor reactivity impairment, insulin resistance, inflammation, oxidative stress, platelet activation and hypercoagulation) have been proposed [15, 20–24]. Under the etiopathology, atherothrombotic stroke may suddenly occur in multiple lesions, and/or the repeated stroke formation may be unnoticed in some cases whose lesions are multiple, like a silent brain infarct [25, 26]. Gender differences exist in the clinical features of MetS, stroke and their association thereof [11, 27–29]; that is, the influences of MetS on stroke may be greater in Japanese women than in men [27, 28]. According to these literature findings, the associations among MetS and atherothrombotic stroke multiplicity observed in the present survey appear to be plausible. On the other hand, though the impact of MetS on multiple lesions in men was not significant and weaker than that in women, the OR level was over 1.5 (Table 2) and confirmation may be repeatedly required in men. Mutifaceted studies including etiopathological factors and larger samples in both genders will be necessary to understand the findings.

The clinical significance of MetS or each component of MetS on the atherosclerotic outcomes is poorly understood [30–32]. In the present study, the analysis of each component of MetS with multiple lesions showed that dyslipidemia in particular (such as high TG and/or low HDL-C [33]) was positively associated with multiple lesions in both genders. Such a significant influence of dyslipidemia on stroke can be supported by previous studies [11, 34], although these studies did not examine stroke multiplicity. In addition to the treatment of MetS, a consideration of atherogenic dyslipidemia [33] could be particularly pivotal when subjects do not necessarily manifest a complete MetS phenotype.

There are several limitations associated with this study. The study had a retrospective design based on clinical data obtained from medical records. Although the multiple lesions detected in the present study could include old infarcts, the clear discrimination between new and old infarcts was not always made (it was difficult to discriminate them based on a retrospective description of medical records). The number of multiple lesions may also be useful information; however, it could not completely be collected in this survey. The additional information (e.g., serum low-density lipoprotein cholesterol, smoking and alcohol intake) was not fully collected for all patients. Information bias may therefore affect the study results. The study was conducted in a single hospital, which could lead to selection bias. Furthermore, there may be ethnic disparities in the relevance of MetS on stroke multiplicity [10] and the definition of MetS itself may affect the results [14, 35]. These issues should be addressed in future studies with prospective and multicentric designs with various populations.

In summary, MetS may have a pathophysiology associated with intracranial atherothrombotic stroke multiplicity in women in particular. Future studies are warranted to confirm the findings.
